# Spatial Analysis of Access to Psychiatrists for US Military Personnel and Their Families

**DOI:** 10.1001/jamanetworkopen.2022.49314

**Published:** 2023-01-03

**Authors:** Marigee Bacolod, Jennifer Heissel, Yu-Chu Shen

**Affiliations:** 1 Department of Defense Management, Naval Postgraduate School, Monterey, California; 2 National Bureau of Economic Research, Cambridge, Massachusetts

## Abstract

**Importance:**

Military service members and their families have greater mental health care needs compared with their civilian counterparts. Some communities have inadequate access to psychiatrists for this population.

**Objectives:**

To identify geographic variations in the availability of military and civilian psychiatrists within a 30-minute driving time of TRICARE (the US military’s health care program) beneficiaries’ communities and compare the likelihood of living in areas with inadequate access to psychiatrists for historically underserved and other communities.

**Design, Setting, and Participants:**

This retrospective cohort study of all zip code communities in the continental US, Hawaii, and Alaska with at least one TRICARE beneficiary between January 1, 2016, and September 30, 2020, combines data from the Defense Health Agency, the National Plan and Provider Enumeration System, and the US Census to estimate a logistic regression to compare differences between communities with and without a psychiatrist shortage. Data were analyzed from September 2021 to November 2022.

**Main Outcomes and Measures:**

A community’s likelihood of having a shortage of military and civilian psychiatrists within a 30-minute driving time and a community’s likelihood of having no psychiatrists. Odds ratios were calculated to estimate likelihood of the outcomes.

**Results:**

This study includes 39 487 unique communities where 13% of the population is Black and 14% of the population is Hispanic. During the study period, 35% of TRICARE beneficiaries lived in communities with a shortage of both military and civilian psychiatrists, and 6% lived in communities with no access to military or civilian psychiatrists. Low-income communities with high income inequality were 1.64 (95% CI, 1.30-2.07) times more likely to have inadequate access to psychiatrists and 2.59 (95% CI, 1.82-3.69) times more likely to have no access to psychiatrists, compared with reference communities (average income without high income inequality); low-income communities without high income inequality were 1.37 (95% CI, 1.05-1.78) times more likely to have inadequate access to psychiatrists and 1.93 (95% CI, 1.28-2.89) times more likely to have no access to psychiatrists. Rural communities were 6.65 (95% CI, 5.09-8.69) times more likely to have inadequate access to psychiatrists than urban communities.

**Conclusions and Relevance:**

In this cohort study of US communities, 35% of TRICARE beneficiaries lived in communities with inadequate access to psychiatrists. Psychiatric capacity was structurally inequitable along 2 separate dimensions: the income gradient and rurality. Developing targeted strategies for these shortage areas could alleviate disparities.

## Key Points

**Question:**

What is the geographic variation in the capacity of military and civilian psychiatrists within a 30-minute driving time of TRICARE (the US military’s health care program) beneficiaries’ communities, and how does a community’s likelihood of having a psychiatrist shortage differ between historically underserved and other communities?

**Findings:**

In this cohort study of 39 487 zip code communities between 2016 and 2020, 35% of TRICARE beneficiaries lived in communities with a shortage of both military and civilian psychiatrists and 6% of beneficiaries had no psychiatrists available within a 30-minute driving time. Beneficiaries in low-income communities with high income inequality and rural communities had the highest likelihood of experiencing a shortage of psychiatrists.

**Meaning:**

The study suggests that rural and economically disadvantaged communities need targeted strategies as the US Department of Defense considers realignment of military psychiatric capacity, because they cannot rely on civilian psychiatrists to fulfill the need gap for these shortage areas.

## Teaser

This cohort study identifies geographic variations in the availability of military and civilian psychiatrists within a 30-minute driving time of TRICARE beneficiaries’ communities and compares the likelihood of living in areas with inadequate access to psychiatrists for underserved and other communities.

## Introduction

Structural barriers to care for the US population are particularly salient in mental health settings, including long waiting lists, clinicians not accepting new patients, and a lack of insurance coverage.^1^ Given military provision of care, it is less clear whether military-affiliated populations are similarly affected by access inequity, especially in historically underserved areas. Military service members and their families generally have higher mental health care needs than the civilian population, and they also have unique challenges to access mental health care services due to military deployments, other high-risk and stressful activities associated with their jobs, frequent relocations, and family member absences.^2,3^ Although many active duty service members and some military families live on or near military installations and use military treatment facilities (MTFs) for medical care, many military families rely on civilian medical professionals.^3^

Geographic variation in health care spending, use, and quality has been well documented in the civilian market, and there is continued debate about whether resources are efficiently allocated across space.^4,5,6,7,8^ Although some geographic variation in health care spending and use might be due to individual clinician or patient behaviors, the built environment of health care supply also contributes to access and treatment inequities for historically underserved populations.^9,10^ The per-capita supply of behavioral health clinicians varies substantially across Census divisions and between urban and rural areas,^11^ and specialized services such as percutaneous coronary intervention facilities or stroke care are underprovided in historically underserved (such as low-income or racially segregated) communities while oversupplied in other communities.^12,13^ The military health system, despite being centrally managed, can still experience unexplained geographic variation in spending and use of selective procedures.^14,15^ Beneficiaries of TRICARE (the US military’s health care program) have similar geographic variations in health care use as the colocated civilian population for primary care, but less variation for specialty care.^16^ Military treatment facilities provide an alternative to civilian health care resources (and vice versa), which could reduce disparities for beneficiaries who live in historically underserved communities. However, to our knowledge, little research exists examining the simultaneous psychiatric capacity of MTFs and civilian facilities for the military population.

Our study has 2 objectives. First, we analyze geographic variations in psychiatric capacity within 30-minute driving times of TRICARE beneficiaries’ communities, focusing on availability of both MTF and civilian psychiatrists. Second, we compare the likelihood of living in areas with psychiatrist shortages between beneficiaries in historically underserved communities (as measured by socioeconomic condition, rurality, and racial and ethnic distribution) and other communities. We hypothesize that historically underserved communities are more likely to have psychiatrist shortages.

## Methods

### Study Population

The study population includes 39 487 zip code communities in the United States (excluding Puerto Rico and US territories) that contained at least 1 TRICARE beneficiary between January 1, 2016, and September 30, 2020. Communities are defined by beneficiary mailing zip code. This study is approved through the Naval Postgraduate School institutional review board and follows the Strengthening the Reporting of Observational Studies in Epidemiology (STROBE) reporting guideline. Because the data were administratively collected and deidentified, the study did not require informed consent.

### Data Sources

We combined monthly data to form our analysis to measure beneficiary population size, demographic, and military branch of service characteristics of TRICARE beneficiaries; civilian and MTF psychiatrist capacity; and community characteristics. Data sources include data from the Defense Health Agency, the National Plan and Provider Enumeration System National Provider Identifier data, the US Census, American Community Survey, and the Social Determinants of Health Database.^17^ We used a web-based query^18^ to derive a database of driving time between centers of each zip code community and (1) the practice location of the MTFs’ psychiatrists and (2) the zip code centers of civilian psychiatrists’ practice location. See eAppendix 1 in Supplement 1 for details.

### Defining Relevant Geographic Access Area From the Patient Perspective

We used zip codes to define community because it is the smallest geographic unit we can capture across data sources. We used travel time from the patient’s perspective to define the relevant access area, rather than political boundaries (eg, county lines) or a fixed radius approach (eg, 20-mile radius). Each mental health episode can require multiple follow-up visits, so we chose a 30-minute car driving time (1-hour round trip) as a reasonable threshold for TRICARE beneficiaries to travel on a repeated basis. Although driving time accounts for a large percentage of travel time, actual travel time would be longer than 30 minutes (eg, navigating parking, waiting for a ride, stops on public transportation). We provide additional rationale for our geographic definition in eAppendix 1 in Supplement 1.

### Outcomes

Our primary outcome is whether a community is a psychiatrist shortage area. The Health Resources and Services Administration (HRSA) defines shortage areas as those where the population-to-psychiatrist ratio exceeds 20 000:1 for populations with unusually high needs of mental health care and 30 000:1 for populations with normal needs.^19^ We used the 20 000:1 threshold because the military population has higher exposure to stress and risks. We created separate shortage area designators for MTF-provided care and care from civilian facilities because the relevant populations are different. For MTF-provided care, the relevant population is TRICARE beneficiaries because non-TRICARE patients cannot use the MTF, and the ratio was computed as the number of full-time equivalent psychiatrists in the MTF within a 30-minute driving time of a given community divided by the number of TRICARE beneficiaries within a 30-minute driving time. For civilian psychiatrists, the relevant patient population is all residents, regardless of insurance status. We computed the ratio as the number of civilian psychiatrists practicing in zip codes within a 30-minute driving time of a given community divided by the number of residents within the same set of zip codes. A community was considered a shortage area for both military and civilian psychiatrist capacity if both ratios were smaller than 1:20 000. Our secondary outcome is a subset of a shortage area that has no military or civilian psychiatrists available within a 30-minute driving time.

### Statistical Analysis

Our unit of analysis was the community-month. Because our outcomes are binary, we implemented logistic regressions in Stata, version 17 (StataCorp LLC),^20^ with robust SEs that accounted for 2 levels of correlations: intracommunity correlation among observations from different months of the same community and intercommunity correlations among communities from the same county, as those communities might be subject to similar local health regulations. All regressions were weighted by the beneficiary population size of each community to obtain TRICARE beneficiary-representative estimates.^21^ All models included time dummies to control for macro trends. All *P* values were from 2-sided tests and results were deemed statistically significant at *P* < .05.

We began with a set of single-dimension models, where we estimated logistic models separately for each set of dimensions. The single-dimension model estimated the overall variation of each dimension, without accounting for underlying differences in other dimensions that could act as variance mediators. The first 3 models captured communities with a high share of underserved populations (based on definitions used by the National Institutes of Health US health disparity populations).^22^ Model 1 focused on socioeconomic conditions based on income level (as measured by median family income) and income inequality (as measured by the Gini coefficient [eAppendix 2 in Supplement 1]). A Gini value of 0 means that everyone has the same amount of income and a Gini value of 1 means that 1 household has all the income. Using both income level and income inequality allowed us to capture a more complete picture of a community’s economic condition, as a community might be classified as mean income but actually have a highly bimodal income distribution. Specifically, we classified communities based on tertiles of their median family income per the US Census (low-, average-, and high-income communities) and by whether their Gini coefficient was in the upper one-third of the overall Gini coefficient distribution. We classified each community into 1 of 6 categories: mean income without high income inequality (reference group), mean income with high income inequality, low income without high income inequality, low income with high income inequality, high income without high income inequality, and high income with high income inequality.

Model 2 focused on regional variations and included indicators for geographic regions (South [reference group], Midwest, Northeast, West, Alaska, Hawaii) and whether the zip code community was defined as rural by the Federal Office of Rural Health Policy.^23^ Model 3 focused on racial and ethnic distribution of the communities and included 2 sets of categorical variables based on their Black and Hispanic share of population: whether a community is in the lowest (reference group), middle, or highest tertile of Black residents and likewise for Hispanic distribution. Race and ethnicity information were self-identified by respondents to the US Census.

Model 4 focused on service branch distribution for a given community. A community was classified as an Army community if at least 50% of beneficiaries in that community were affiliated with the Army, and likewise for the Navy, Air Force, and Marine Corps. Communities where no service exceeded 50% were classified as Joint (reference group). Model 5 focused on size and type of TRICARE beneficiaries in each community. We categorized a community as having low, medium, or high presence of TRICARE based on tertile of the share of the community population that are TRICARE beneficiaries. We further captured the presence of different types of TRICARE beneficiaries based on tertile of the share of TRICARE beneficiaries who were dependent children, retirees, or other nonactive duty population. Dependent children are children of active duty primary beneficiaries, while retirees have retired from a military career, often after at least 20 years of service.^24^ Other included nonactive duty family members such as spouses of the active duty beneficiaries.^25^

Finally, model 6 was a fully adjusted model that included the complete set of factors from models 1 to 5 to estimate the association between a community’s psychiatrist shortage status and a single factor, after accounting for all other factors included in the model.

## Results

Table 1 shows community characteristics of the 39 487 communities included in the study, overall and by whether we defined the community as a psychiatrist shortage area. A total of 35% of TRICARE beneficiaries resided in communities with a shortage of both MTF and civilian psychiatrists; 6% had no psychiatrist of either type within a 30-minute travel time. Most TRICARE beneficiaries resided in the South Census region (55%), and 33% were in communities classified as mean income without high income inequality. The mean TRICARE beneficiary lives in an area where 13% of the full population identified as Black and 14% identified as Hispanic. The Army was the most common service affiliation (mean [SE], 41% [29%]), and the TRICARE beneficiary population was 15% (30%) active duty, 21% (11%) dependent children, 24% (13%) retirees, and 40% (14%) other.

**Table 1.** Summary Statistics of TRICARE Beneficiary Distribution by MTF and Civilian Psychiatrist Shortage Areas^a^

Characteristic	Mean (SD), %^b^			Difference (*P* value)
	All	Shortage of MTF and civilian psychiatrists	All other communities	
Capacity measures				
Shortage area for both MTF and civilian psychiatrists	35 (46)	100 (0)	0 (0)	NA
Area with no MTF or civilian psychiatrists within 30-min drive	6 (24)	20 (40)	0 (1)	0.20 (<.001)
Census regions				
Alaska	1 (10)	1 (9)	1 (10)	−0.00 (<.001)
Hawaii	2 (13)	1 (9)	2 (15)	−0.01 (<.001)
Midwest	12 (33)	13 (33)	12 (33)	0.01 (<.001)
Northeast	7 (26)	4 (19)	9 (28)	−0.05 (<.001)
South	55 (50)	61 (49)	52 (50)	0.09 (<.001)
West	23 (42)	21 (40)	24 (43)	−0.03 (<.001)
Rural	16 (37)	32 (47)	9 (29)	0.22 (<.001)
Census socioeconomic characteristics				
Median (IQR) family income, $	27 167 (22 370-33 861)	26 005 (21 919-31 241)	27 966 (22 658-36 072)	−1961 (<.001)
Gini coefficient	0.41 (0.06)	0.42 (0.05)	0.40 (0.06)	0.02 (<.001)
Low income without high income inequality	27 (45)	29 (46)	26 (44)	0.03 (<.001)
Low income with high inequality	7 (25)	12 (32)	4 (20)	0.07 (<.001)
Mean income without high inequality	33 (47)	33 (47)	33 (47)	0.00 (<.001)
Mean income with high inequality	6 (24)	8 (27)	5 (22)	0.03 (<.001)
High income without high inequality	21 (41)	15 (35)	24 (42)	−0.09 (<.001)
High income with high inequality	7 (25)	4 (18)	8 (27)	−0.04 (<.001)
Census demographic characteristics				
Total population, No.	27 756.17 (19 702.98)	32 612.44 (20 564.55)	25 452.55 (18 848.27)	7159.89 (<.001)
Black	13 (15)	13 (15)	13 (15)	0.00 (<.001)
Hispanic	14 (17)	14 (19)	14 (16)	−0.00 (<.001)
Beneficiary service affiliation				
Army	41 (29)	45 (28)	39 (29)	0.06 (<.001)
Navy	21 (24)	19 (20)	22 (25)	−0.03 (<.001)
Marine Corps	8 (15)	7 (14)	8 (16)	−0.00 (<.001)
Air Force	27 (25)	25 (22)	28 (26)	−0.03 (<.001)
Beneficiary type				
Active duty	15 (30)	7 (17)	19 (33)	−0.12 (<.001)
Children	21 (11)	23 (10)	20 (12)	0.02 (<.001)
Military retirees	24 (13)	28 (11)	23 (14)	0.05 (<.001)
Other nonactive duty beneficiaries	40 (14)	43 (9)	38 (16)	0.05 (<.001)
No. of community-months	2 098 124	731 970	1 366 154	NA
No. of unique communities	39 487	14 206	26 820	NA
No. of beneficiaries (monthly mean)	8 942 401	2 823 761	6 118 640	NA

Abbreviations: MTF, military treatment facility; NA, not applicable.

^a^ TRICARE is the US military’s health care program.

^b^ Columns 2 to 4 report means and SDs at zip code level, weighted by beneficiary population. Column 5 reports mean difference of columns 3 and 4 and reports *P* values of the *t* test of mean differences in parentheses. Clinician counts are top-coded at the 99th percentile.

**Figure 1.**
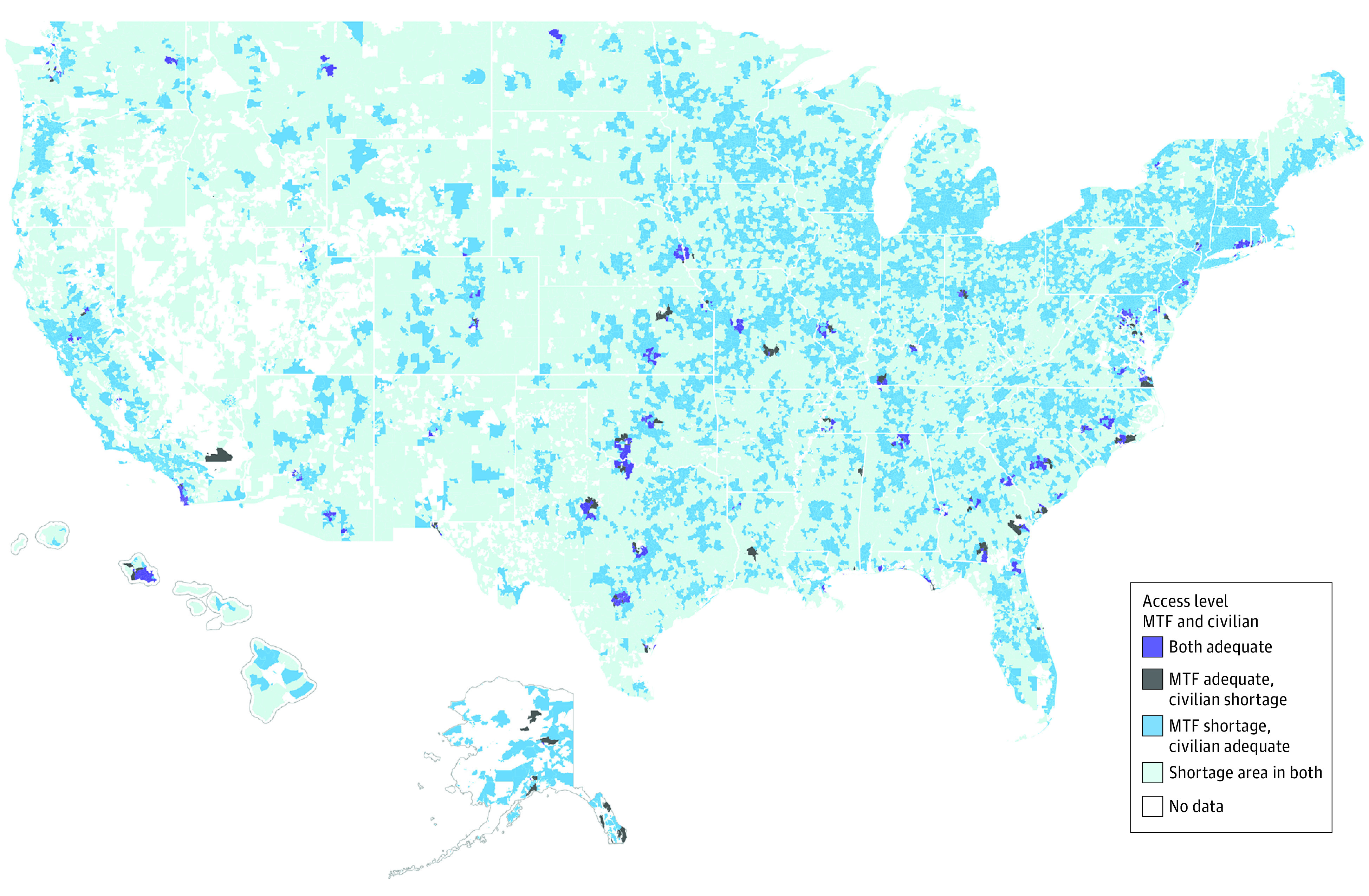
Access to Military Treatment Facility (MTF) and Civilian Psychiatrists Within 30-Minute Driving Time in 2019

Figure 1 shows the geographic distribution of communities by 4 levels of access to psychiatrists. The purple pockets contain 13% of the TRICARE beneficiary population and represent communities with an adequate number of psychiatrists in both MTF and civilian facilities. The gray pockets, with 5% of beneficiaries, have an adequate number of psychiatrists in an MTF but have a shortage of civilian psychiatrists. About 47% of TRICARE beneficiaries reside in dark blue communities, notably in the Northeast and coastal West regions, with adequate civilian psychiatrists but a shortage of MTF psychiatrists. Finally, 35% of beneficiaries reside in light blue communities, classified as shortage areas in both the MTF and civilian sectors, mainly in the South, interior West, and Midwest regions. The fraction of TRICARE beneficiaries in shortage areas remain similar over time. Additional details about temporal trends are included in eAppendix 3 Supplement 1.

**Figure 2.**
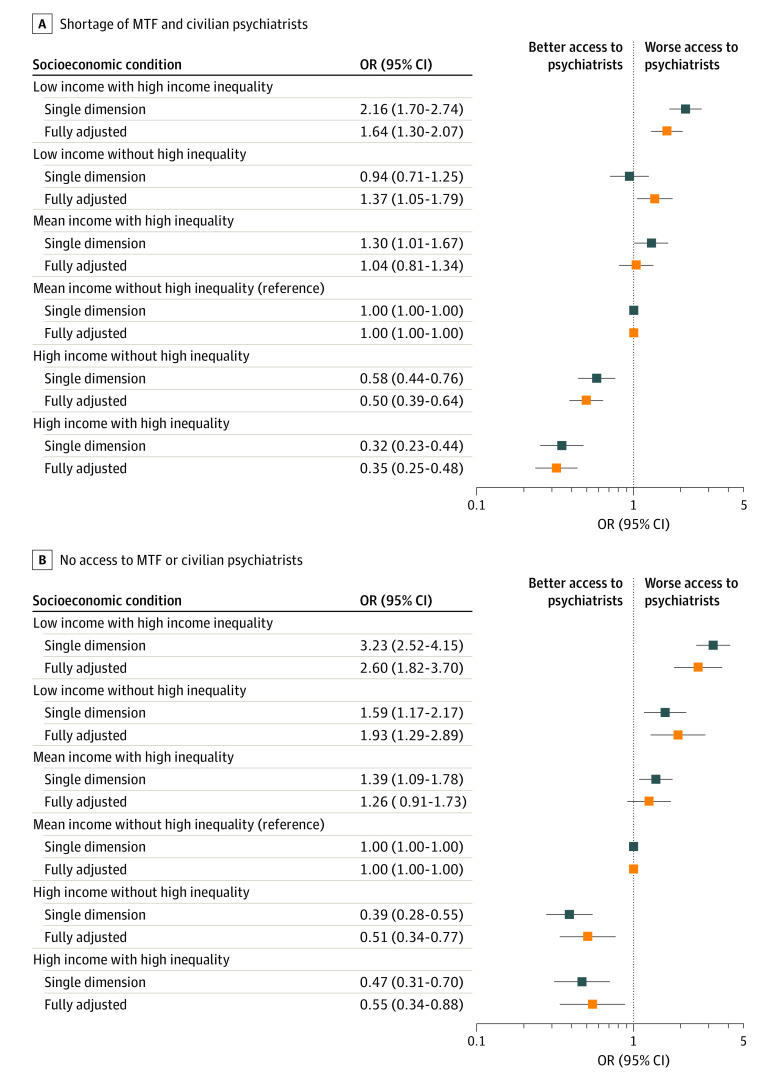
Logistic Regression Odds Ratios (ORs) of Economic Status for Access to Psychiatrists Within 30-Minute Drive Robust SEs clustered at zip code and county levels. Regressions weighted by beneficiary population. Shortage defined as fewer than 1 psychiatrist per 20 000 relevant population. Other covariates in adjusted models: year, rurality, regions, beneficiary and demographic characteristics, total population. MTF indicates military treatment facility.

Figure 2 and the first panel of Table 2 reveal that communities classified as both low income and high income inequality had the highest odds of having a psychiatrist shortage—they were 2.16 (95% CI, 1.70-2.74) times more likely to be designated a shortage community compared with communities with mean income and without high income inequality in the single dimension model (the reference community). The odds remained high (odds ratio [OR], 1.64; 95% CI, 1.30-2.07) after controlling for other community characteristics in model 6. Low-income communities without high income inequality were similar to the reference community in the single dimension model (OR, 0.94; 95% CI, 0.71-1.25), but were 1.37 (95% CI, 1.05-1.78) times more likely to be a shortage area in the fully adjusted model. In the fully adjusted model, communities with high income had lower odds of being designated as a shortage area whether they had high income inequality (OR, 0.32; 95% CI, 0.23-0.44) or did not have high income inequality (OR, 0.50; 95% CI, 0.39-0.64).

**Table 2.** Logistic Regression Odds Ratios for Access to Psychiatrists Within 30-Minute Drive

Model^a^	Odds ratio (95% CI)			
	Shortage area for both MTF and civilian psychiatrists		No MTF or civilian psychiatrists within 30-min drive	
	Single dimension models	Model 6: fully adjusted	Single dimension models	Model 6: fully adjusted
**Model 1: socioeconomic conditions**				
Mean income without high income inequality	1 [Reference]	1 [Reference]	1 [Reference]	1 [Reference]
Low income with high income inequality	2.16 (1.70-2.74)^b^	1.64 (1.30-2.07)^b^	3.23 (2.52-4.15)^b^	2.59 (1.82-3.69)^b^
Low income without high income inequality	0.94 (0.71-1.25)	1.37 (1.05-1.78)^b^	1.59 (1.17-2.17)^b^	1.93 (1.28-2.89)^b^
Mean income with high income inequality	1.30 (1.01-1.67)^c^	1.04 (0.81-1.34)	1.39 (1.09-1.78)^b^	1.26 (0.91-1.73)
High income without high income inequality	0.58 (0.44-0.76)^b^	0.50 (0.39-0.64)^b^	0.39 (0.28-0.55)^b^	0.51 (0.34-0.77)^b^
High income with high income inequality	0.35 (0.25-0.48)^b^	0.32 (0.23-0.44)^b^	0.47 (0.31-0.70)^b^	0.55 (0.34-0.88)^c^
**Model 2: regions and rurality**				
Community				
Urban	1 [Reference]	1 [Reference]	1 [Reference]	1 [Reference]
Rural	4.35 (3.39-5.59)^b^	6.65 (5.09-8.69)^b^	14.05 (10.14-19.48)^b^	5.48 (4.15-7.25)^b^
South	1 [Reference]	1 [Reference]	1 [Reference]	1 [Reference]
Alaska	0.48 (0.22-1.08)	0.68 (0.34-1.38)	3.81 (1.10-13.26)^c^	7.65 (1.72-34.09)^b^
Hawaii	0.21 (0.16-0.28)^b^	0.16 (0.10-0.24)^b^	2.43 (1.90-3.10)^b^	3.49 (1.75-6.97)^b^
Midwest	0.60 (0.45-0.79)^b^	0.51 (0.37-0.70)^b^	0.78 (0.55-1.11)	0.57 (0.41-0.79)^b^
Northeast	0.31 (0.23-0.43)^b^	0.34 (0.25-0.46)^b^	0.51 (0.34-0.75)^b^	0.32 (0.22-0.45)^b^
West	0.68 (0.45-1.01)	0.61 (0.36-1.01)	1.75 (1.19-2.58)^b^	2.31 (1.56-3.41)^b^
**Model 3: race and ethnicity distribution**				
Share of population that are Black				
Low	1 [Reference]	1 [Reference]	1 [Reference]	1 [Reference]
Medium	0.79 (0.65-0.97)^c^	0.80 (0.65-0.98)^c^	0.44 (0.31-0.62)^c^	0.65 (0.43-0.99)^c^
High	0.79 (0.60-1.05)	0.54 (0.38-0.78)^b^	0.32 (0.23-0.44)^b^	0.50 (0.35-0.71)^b^
Share of population that are Hispanic				
Low	1 [Reference]	1 [Reference]	1 [Reference]	1 [Reference]
Medium	0.76 (0.63-0.93)^b^	1.00 (0.83-1.22)	0.73 (0.57-0.94)^c^	0.89 (0.68-1.16)
High	0.63 (0.46-0.86)^b^	0.68 (0.45-1.04)	0.70 (0.45-1.10)	0.75 (0.47-1.19)
**Model 4: primary service branch of beneficiaries**				
Mixed service community	1 [Reference]	1 [Reference]	1 [Reference]	1 [Reference]
Army	1.16 (0.92-1.47)	1.08 (0.86-1.37)	1.21 (0.91-1.60)	1.16 (0.93-1.45)
Navy	0.58 (0.30-1.14)	0.70 (0.35-1.37)	0.34 (0.18-0.61)^b^	0.52 (0.28-0.97)^c^
Marine Corps	0.56 (0.33-0.95)^c^	0.59 (0.26-1.31)	0.43 (0.17-1.08)	0.38 (0.07-2.15)
Air Force	0.68 (0.50-0.94)^c^	0.80 (0.53-1.21)	0.67 (0.42-1.04)	0.70 (0.38-1.29)
**Model 5: presence and type of beneficiaries**				
Share of population that are covered by TRICARE^d^				
Low	1 [Reference]	1 [Reference]	1 [Reference]	1 [Reference]
Medium	1.05 (0.83-1.32)	1.27 (1.00-1.62)^c^	0.94 (0.77-1.15)	1.30 (1.06-1.60)^c^
High	0.67 (0.47-0.97)^c^	0.90 (0.59-1.38)	0.58 (0.33-1.02)	0.67 (0.29-1.55)
Share of TRICARE that are dependent children				
Low	1 [Reference]	1 [Reference]	1 [Reference]	1 [Reference]
Medium	1.33 (1.06-1.67)	1.14 (0.92-1.42)	0.55 (0.45-0.66)^b^	1.08 (0.89-1.31)
High	1.75 (1.33-2.31)^b^	1.29 (0.95-1.77)	0.93 (0.59-1.46)	2.42 (1.35-4.33)^b^
Share of TRICARE that are retirees				
Low	1 [Reference]	1 [Reference]	1 [Reference]	1 [Reference]
Medium	1.42 (1.00-2.03)	1.52 (1.03-2.25)^c^	1.61 (0.90-2.86)	2.13 (1.02-4.44)^c^
High	1.96 (1.37-2.81)^b^	1.92 (1.28-2.89)^b^	4.20 (2.64-6.69)^b^	3.83 (1.85-7.93)^b^
Share of TRICARE that are other nonactive duty				
Low	1 [Reference]	1 [Reference]	1 [Reference]	1 [Reference]
Medium	1.21 (0.91-1.60)	0.93 (0.70-1.23)	0.60 (0.48-0.76)^b^	1.09 (0.78-1.51)
High	1.00 (0.75-1.32)	0.66 (0.50-0.86)^b^	0.56 (0.46-0.68)^b^	1.10 (0.86-1.39)
No. of community-years	2 098 124			

Abbreviation: MTF, military treatment facility.

^a^ Additional variables common in all models include year dummies and community population (log transformed). Robust SEs are clustered at zip code community and county levels.

^b^ *P* < .01.

^c^ *P* < .05.

^d^ TRICARE is the US military’s health care program.

There were larger disparities across income gradiant when the outcome is a community have no access to a psychiatrist instead of having a shortage of psychiatrists. In the fully adjusted model, communities with low income and high income inequality had the highest odds of having no access to psychiatrists (OR, 2.59; 95% CI, 1.82-3.69), followed by low-income communities without high income inequality (OR, 1.93; 95% CI, 1.28-2.89) (Table 2). High-income communities had lower odds of no access (without high income inequality: OR, 0.51; 95% CI, 0.34-0.77; with high income inequality: OR, 0.55; 95% CI, 0.34-0.88).

The second panel of Table 2 shows regional differences; we focus on the fully adjusted model for the remainder of the results. Consistent with prior literature,^26,27,28,29^ rural communities were 6.65 (95% CI, 5.09-8.69) times more likely than urban communities to have a shortage of both MTF and civilian psychiatrists and 5.48 (95% CI, 4.15-7.25) times as likely to have no psychiatrists. The patterns demonstrated that the South was most likely to have a shortage compared with the other areas, but Alaska (OR, 7.65; 95% CI, 1.72-34.09) and Hawaii (OR, 3.49; 95% CI, 1.75-6.97) were most likely to have no psychiatrists.

The third panel of Table 2 shows that communities with a high share of individuals from racial or ethnic minority groups had lower odds of being in psychiatrist shortage areas. For example, communities with a high share of Black individuals were less likely (OR, 0.54; 95% CI, 0.38-0.78) than communities with a low share to have no access to psychiatrists within 30 minutes, perhaps because Black individuals tend to be concentrated in urbanized communities.

After accounting for other factors, service affiliation was not associated with being in a shortage area. Communities with a high share of TRICARE retirees were 1.92 (95% CI, 1.28-2.89) times more likely to be in shortage areas and 3.83 (95% CI, 1.85-7.93) times more likely to have no access to psychiatrists compared with communities with a low presence of military retirees (Table 2).

We conducted several sensitivity analyses. First, we redefined *shortage area* as a 30 000:1 population-to-psychiatrist ratio. Under this definition, 24% of TRICARE beneficiaries lived in communities with a psychiatrist shortage. Second, we expanded drive time to 40 minutes. Under this definition, 27% of TRICARE beneficiaries lived in communities with a psychiatrist shortage. Finally, less than 40% of behavioral health professionals (including psychiatrists) accepted new TRICARE patients between 2008 and 2020.^30,31^ Using fiscal year 2021 as the benchmark, if we assume that only 37% of civilian psychiatrists were accessible to TRICARE beneficiaries, 60% of TRICARE beneficiaries lived in communities with a psychiatrist shortage. Logistic regression results of community characteristics remained similar across all 3 alternative assumptions (eTables 1-3 in Supplement 1).

## Discussion

Our analysis of psychiatrist capacity for TRICARE beneficiaries between January 2016 and September 2020 shows that 35% of TRICARE beneficiaries live in communities with a shortage of both MTF and civilian psychiatrists and 6% have no psychiatrists from either sector within a 30-minute driving time. These beneficiaries are concentrated in economically disadvantaged or rural communities. Specifically, access to psychiatrists is progressively worse as we move down the income gradient: those with low income and high income inequality have the highest likelihood of a psychiatrist shortage or no access to psychiatrists, followed by communities that are low income but without high income inequality. High-income communities (regardless of income inequality) have the lowest likelihood of experiencing psychiatrist shortage.

Our findings reveal important disparities in access to psychiatrists among military retirees. Military retirees are especially vulnerable to mental health problems.^29,32,33^ However, our findings show that military retirees are more likely to be in communities with too few or no psychiatrists. Although the access gap for this subgroup might be filled partially by care provided by the Veterans Administration (VA), only 60% of veterans are eligible for VA health care under the current policy and only 50% of eligible veterans use VA health benefits.^34,35^

Providing mental health care to the military population and their dependents involves multiple health care systems—TRICARE, VA, and the private sector. The fact that the military population, despite having access to care in multiple systems, is not immune to structural inequity in psychiatric care provision is perhaps not surprising; there are fewer than 500 MTFs that provide psychiatric care, while the military population is distributed widely over 39 487 zip codes. The military population must rely on civilian psychiatric care in many areas. Consistent with prior studies,^5,6,7,8,26,27,36,37^ our results highlight that market forces incentivize civilian mental health professionals to locate in nonrural and affluent communities, but they also represent opportunities where the public sector can play a role in reducing disparities. As the military health system considers realignment of military psychiatric capacity, it could develop strategies to mitigate disparities between affluent and underserved communities. Such strategies could include optimally placing satellite clinics or coordinating telemedicine capacity supported by MTF psychiatrists. The military health system could alter its reimbursement rates for care purchased from civilian mental health care professionals to mitigate shortage problems in economically underserved and rural communities. However, any solutions must work within the broader context of a general psychiatrist shortage.^7,36,37,38^ The HRSA projects that by 2030 the US will have 12 500 fewer adult psychiatrists than needed.^39^ The HRSA also projects an increase in nurse practitioners, who can also prescribe medication. Future studies should examine whether increasing nurse practitioners in communities with a psychiatrist shortage would effectively improve mental health care needs for the military population.

Although military populations are important in their own right, our analysis also answers the call for increased research regarding structural discrimination and inequity more broadly^40,41,42^ by quantifying geographically based shortages in access to psychiatrists in historically underserved civilian communities.^43,44^ Civilian populations within psychiatrist shortage areas share similar experiences with their military counterparts. Improving the psychiatrist capacity provided by the military health system could reduce shortage problems experienced by civilians, if initiatives free up the resources of civilian psychiatrists. State or federal governments could also decrease disparities within both civilian and military populations by, for instance, providing geographically based incentives in loan repayment programs.

### Limitations

This study includes several limitations. First, while we recognize that psychiatric nurse practitioners and primary care physicians can also prescribe medications and serve in similar roles as psychiatrists, we did not include them in measuring psychiatric capacity, because we needed to be consistent with the HRSA population-to-psychiatrist definition of *shortage*. Data on psychiatrists are also more reliable than the broader set of mental health care professionals, especially in the civilian data. The focus on psychiatrists reduces estimation errors.

Second, our capacity metrics are measured with error if there are registry processing delays when a practitioner moves or closes a clinic. If these errors do not systematically vary by community characteristics, it should not confound our findings. Driving time was also measured with error because the query assumes normal traffic. Both sources of measurement error create attenuation bias, making our estimated results conservative or lower than they should be.

Third, we focused on access barriers of the built environment. A military-affiliated individual with psychiatrists available within a 30-minute travel time can still face many other barriers to accessing psychiatric care, including difficulty in getting a referral, lack of coordination among primary care and specialists, office visit wait times, and waiting lists for new patients.^30,45^

Fourth, not all civilian psychiatrists accept TRICARE patients due to low reimbursements. When we assumed a uniform 37% acceptance rate across all communities (per fiscal year 2021^31^), the percentage of TRICARE beneficiaries living in communities with a psychiatrist shortage increased to 60%. However, if acceptance rates are lower in historically underserved communities, we would underestimate the true disparity between historically underserved and other communities.

Fifth, many communities have a shortage of MTF psychiatrists but adequate access to civilian psychiatrists. Although psychiatrists working in MTFs and civilian practices underwent similar training, substituting a psychiatrist well acquainted with military issues with a psychiatrist in civilian practice may not be an equal swap.

Sixth, we used a 30-minute driving time as the geographic access area to identify shortages, following Centers for Medicare & Medicaid Services and HRSA guidelines. This definition might result in overestimating the population-to-psychiatrist ratio in urbanized areas where both population and psychiatrists are dense. This might exacerbate the estimated access disparity between urban and rural regions, where population and psychiatrists are dispersed. However, sensitivity analyses showed that results were similar when we changed our shortage threshold from a ratio of 20 000:1 to 30 000:1, changed drive time to 40 minutes, or specified as outcome having no psychiatrist within the drivable area. Driving is also only part of travel time—actual travel time would be longer, making our estimates conservative. Overall, the purpose of this article is not to estimate the precise number of psychiatrists the TRICARE population have access to, but rather to identify characteristics associated with shortage areas so that decision makers can target resources accordingly.

## Conclusions

In this cohort study of US zip code communities, 35% of TRICARE beneficiaries lived in communities with a shortage of psychiatrists from both MTF and civilian sectors, and 6% had no psychiatrists available within a 30-minute driving time. Beneficiaries living in low-income communities with high income inequality and rural communities had the highest likelihood of experiencing a shortage of psychiatrists from both the military and civilian sectors. Communities with a higher presence of military retirees also had higher risks of experiencing a shortage of or no access to psychiatrists. Our findings suggest that psychiatric capacity was structurally inequitable along the income gradient and rurality and that certain types of TRICARE beneficiaries had worse access than others. As the military health system considers realignment of its psychiatric capacity, it would be important to develop targeted strategies for shortage areas, since it cannot rely on civilian mental health care professionals to care for the military population in many communities.

## Notes

Supplement 1. **eAppendix 1.** Data and Methods to Define Relevant Geographic Access Area From Patient Perspective **eAppendix 2.** Measurement of Gini Coefficient to Capture Income Inequality **eAppendix 3.** Time Trends
eReferences
**eTable 1.** Logistic Regression Results Where Shortage Is Defined as <1 Psychiatrist per 30K Relevant Population **eTable 2.** Logistic Regression Assuming Relevant Geographic Access Area Is Within 40-Min Drive **eTable 3.** Logistic Regression Assuming Only 37% of Civilian Providers Accept TRICARE

Supplement 2.
Data Sharing Statement

## References

[1] National Alliance on Mental Illness. The doctor is out: continuing disparities in access to mental and physical health care. November 2017. Accessed November 8, 2022. https://www.nami.org/Support-Education/Publications-Reports/Public-Policy-Reports/The-Doctor-is-Out/DoctorIsOut

[2] Fikretoglu D, Sharp ML, Adler AB, . Pathways to mental health care in active military populations across the Five-Eyes nations: an integrated perspective. Clin Psychol Rev. 2022;91:102100. doi:10.1016/j.cpr.2021.102100 34871868

[3] Huebner CR; Section on Uniformed Services; Committee on Psychosocial Aspects of Child and Family Health. Health and mental health needs of children in US military families. Pediatrics. 2019;143(1):e20183258. 30584059 10.1542/peds.2018-3258

[4] Newhouse J, Garber A, Graham R, McCoy M, Mancher M, Kibria A. Interim Report of the Committee on Geographic Variation in Health Care Spending and Promotion of High-Value Care: Preliminary Committee Observations. Institute of Medicine; 2013. 24851296

[5] Smith-East M, Neff DF. Mental health care access using geographic information systems: an integrative review. Issues Ment Health Nurs. 2020;41(2):113-121. doi:10.1080/01612840.2019.1646363 31661647

[6] Hunt JB, Eisenberg D, Lu L, Gathright M. Racial/ethnic disparities in mental health care utilization among U.S. college students: applying the Institution of Medicine definition of health care disparities. Acad Psychiatry. 2015;39(5):520-526. doi:10.1007/s40596-014-0148-1 25026942

[7] Kalb LG, Holingue C, Stapp EK, Van Eck K, Thrul J. Trends and geographic availability of emergency psychiatric walk-in and crisis services in the United States. Psychiatr Serv. 2022;73(1):26-31. doi:10.1176/appi.ps.202000612 34126779 PMC8671549

[8] Thomas KC, Ellis AR, Konrad TR, Holzer CE, Morrissey JP. County-level estimates of mental health professional shortage in the United States. Psychiatr Serv. 2009;60(10):1323-1328. doi:10.1176/ps.2009.60.10.1323 19797371

[9] Alvidrez J, Castille D, Laude-Sharp M, Rosario A, Tabor D. The National Institute on Minority Health and Health Disparities research framework. Am J Public Health. 2019;109(S1):S16-S20. doi:10.2105/AJPH.2018.304883 30699025 PMC6356129

[10] Brown AF, Ma GX, Miranda J, . Structural interventions to reduce and eliminate health disparities. Am J Public Health. 2019;109(S1):S72-S78. doi:10.2105/AJPH.2018.304844 30699019 PMC6356131

[11] Andrilla CHA, Patterson DG, Garberson LA, Coulthard C, Larson EH. Geographic variation in the supply of selected behavioral health providers. Am J Prev Med. 2018;54(6S3):S199-S207. 10.1016/j.amepre.2018.01.004 29779543

[12] Hsia RY, Shen YC. Percutaneous coronary intervention in the United States: risk factors for untimely access. Health Serv Res. 2016;51(2):592-609. doi:10.1111/1475-6773.12335 26174998 PMC4799910

[13] Shen YC, Chen G, Hsia RY. Community and hospital factors associated with stroke center certification in the United States, 2009 to 2017. JAMA Netw Open. 2019;2(7):e197855-e197855. doi:10.1001/jamanetworkopen.2019.7855 31348507 PMC6661722

[14] Kimsey L, Olaiya S, Smith C, . Geographic variation within the military health system. BMC Health Serv Res. 2017;17(1):271. doi:10.1186/s12913-017-2216-1 28407769 PMC5390405

[15] Adesoye T, Kimsey LG, Lipsitz SR, . Geographic variation in Medicare and the military healthcare system. Am J Manag Care. 2017;23(8):e259-e264. 29087149

[16] Bond AM, Schwab SD. Utilization variation in military versus civilian care: evidence from TRICARE. Health Aff (Millwood). 2019;38(8):1327-1334. doi:10.1377/hlthaff.2019.00298 31381387

[17] Agency for Healthcare Research and Quality. Social determinants of health database (beta version). June 2021. Accessed June 29, 2021. https://www.ahrq.gov/sdoh/data-analytics/sdoh-data.html

[18] HERE Developer. Developer guide—HERE routing API. Accessed April 30, 2022. https://developer.here.com/documentation/routing-api/dev_guide/index.html

[19] Health Resources and Services Administration. Designated health professional shortage areas statistics. 2022. Accessed April 27, 2022. https://data.hrsa.gov/Default/GenerateHPSAQuarterlyReport

[20] StataCorp. *Stata Statistical Software: Release 17*. StataCorp LP; 2021.

[21] Stock JH, Watson MW. Heteroskedasticity-robust standard errors for fixed effects panel data regression. Econometrica. 2008;76(1):155-174. doi:10.1111/j.0012-9682.2008.00821.x

[22] National Institute on Minority Health and Health Disparities. Overview. Accessed May 2, 2022. https://www.nimhd.nih.gov/about/overview/

[23] US Health Resources & Services Administration. Federal Office of Rural Health Policy (FORHP) data files. April 28, 2017. Accessed July 1, 2021. https://www.hrsa.gov/rural-health/about-us/what-is-rural/data-files

[24] Military Benefit Association. TRICARE for retirees. September 2021. Accessed November 8, 2022. https://www.militarybenefit.org/membership-benefits/get-educated/tricareforretirees/

[25] TRICARE. Eligibility. March 2, 2022. Accessed November 8, 2022. https://www.tricare.mil/Plans/Eligibility

[26] Morales DA, Barksdale CL, Beckel-Mitchener AC. A call to action to address rural mental health disparities. J Clin Transl Sci. 2020;4(5):463-467. doi:10.1017/cts.2020.42 33244437 PMC7681156

[27] Guerrero APS, Balon R, Beresin EV, . Rural mental health training: an emerging imperative to address health disparities. Acad Psychiatry. 2019;43(1):1-5. doi:10.1007/s40596-018-1012-5 30535843

[28] Kirby JB, Zuvekas SH, Borsky AE, Ngo-Metzger Q. Rural residents with mental health needs have fewer care visits than urban counterparts. Health Aff (Millwood). 2019;38(12):2057-2060. doi:10.1377/hlthaff.2019.00369 31794321

[29] Mott JM, Grubbs KM, Sansgiry S, Fortney JC, Cully JA. Psychotherapy utilization among rural and urban veterans from 2007 to 2010. J Rural Health. 2015;31(3):235-243. doi:10.1111/jrh.12099 25471067

[30] Government Accountability Office. TRICARE multiyear surveys indicate problems with access to care for nonenrolled beneficiaries. Accessed May 23, 2022. https://www.gao.gov/assets/gao-13-364.pdf

[31] TRICARE. TRICARE program effectiveness for FY 2021. Accessed May 23, 2022. https://www.google.com/url?sa=t&rct=j&q=&esrc=s&source=web&cd=&ved=2ahUKEwjfx6n0u_f3AhU5A50JHTTtAcwQFnoECAkQAQ&url=https%3A%2F%2Fhealth.mil%2FReference-Center%2FReports%2F2021%2F07%2F20%2FEvaluation-of-the-TRICARE-Program-FY-2021-Report-to-Congress&usg=AOvVaw18jmrPnw_BBs47NLzxfzl-

[32] National Academies of Sciences, Engineering, and Medicine; Board on Health Care Services; Committee to Evaluate the Department of Veterans Affairs Mental Health Services. Department of Veterans Affairs mental health services: need, usage, and access and barriers to care. In: *Evaluation of the Department of Veterans Affairs Mental Health Services*. National Academies Press; 2018. Accessed May 4, 2022. https://www.ncbi.nlm.nih.gov/books/NBK499497/

[33] Dursa EK, Barth SK, Schneiderman AI, Bossarte RM. Physical and mental health status of Gulf War and Gulf Era veterans: results from a large population-based epidemiological study. J Occup Environ Med. 2016;58(1):41-46. doi:10.1097/JOM.0000000000000627 26716848

[34] US Department of Veterans Affairs. Active-duty service members and VA health care. January 14, 2022. Accessed May 4, 2022. https://www.va.gov/health-care/eligibility/active-duty/

[35] Farmer CM, Hosek SD, Adamson DM. Balancing demand and supply for veterans’ health care: a summary of three RAND assessments conducted under the Veterans Choice Act. Rand Health Q. 2016;6(1):12. 28083440 10.7249/rhq-06-01-12 PMC5158276

[36] Tadmon D, Olfson M. Trends in outpatient psychotherapy provision by U.S. psychiatrists: 1996-2016. Am J Psychiatry. 2022;179(2):110-121. doi:10.1176/appi.ajp.2021.21040338 34875872

[37] McBain RK, Cantor JH, Kofner A, Stein BD, Yu H. Ongoing disparities in digital and in-person access to child psychiatric services in the United States. J Am Acad Child Adolesc Psychiatry. 2022;61(7):926-933. doi:10.1016/j.jaac.2021.11.028 34952198 PMC9209557

[38] McBain RK, Cantor JH, Eberhart NK. Estimating psychiatric bed shortages in the US. JAMA Psychiatry. 2022;79(4):279-280. doi:10.1001/jamapsychiatry.2021.4462 35171208

[39] Health Resources and Service Administration. Behavioral health workforce projections. Accessed November 7, 2022. https://bhw.hrsa.gov/data-research/projecting-health-workforce-supply-demand/behavioral-health

[40] Churchwell K, Elkind MSV, Benjamin RM, ; American Heart Association. Call to action: structural racism as a fundamental driver of health disparities: a presidential advisory from the American Heart Association. Circulation. 2020;142(24):e454-e468. doi:10.1161/CIR.0000000000000936 33170755

[41] Shavers VL, Fagan P, Jones D, . The state of research on racial/ethnic discrimination in the receipt of health care. Am J Public Health. 2012;102(5):953-966. doi:10.2105/AJPH.2012.300773 22494002 PMC3347711

[42] US Department of Health and Human Services. RFA-MD-21-004: Understanding and addressing the impact of structural racism and discrimination on minority health and health disparities (R01 clinical trial optional). Accessed May 28, 2021. https://grants.nih.gov/grants/guide/rfa-files/RFA-MD-21-004.html

[43] Legal Information Institute. 34 US Code §12291—definitions and grant provisions; 2006. Accessed February 18, 2022. https://www.law.cornell.edu/uscode/text/34/12291

[44] US Department of Health and Human Services. *Federal Register*, 42 CFR Parts 5 and 51c, Designation of Medically Underserved Populations and Health Professional Shortage Areas; Proposed Rule; 2008. Accessed February 18, 2022. https://www.federalregister.gov/documents/2008/02/29/E8-3643/designation-of-medically-underserved-populations-and-health-professional-shortage-areas

[45] Doornbos C. Sailors must wait 5 weeks for mental health appointments as Navy battles suicides, top enlisted leader says. *Stars and Stripes*. Accessed May 24, 2022. https://www.stripes.com/branches/navy/2022-05-18/george-washington-aircraft-carrier-sailors-suicides-navy-6049477.html

