# Supplementary material for: Spatial Analysis of Access to Psychiatrists for US Military Personnel and Their Families
Source: JAMA Netw Open. 2023 Jan 3;6(1):e2249314. doi: 10.1001/jamanetworkopen.2022.49314 (PMC9856908; doi:10.1001/jamanetworkopen.2022.49314)
Supplement: Supplement 1. — eAppendix 1. Data and Methods to Define Relevant Geographic Access Area From Patient Perspective eAppendix 2. Measurement of Gini Coefficient to Capture Income Inequality eAppendix 3. Time Trends eReferences eTable 1. Logistic Regression Results Where Shortage Is Defined as <1 Psychiatrist per 30K Relevant Population eTable 2. Logistic Regression Assuming Relevant Geographic Access Area Is Within 40-Min Drive eTable 3. Logistic Regression Assuming Only 37% of Civilian Providers Accept TRICARE [file jamanetwopen-e2249314-s001.pdf]

Supplementary Online Content

Bacolod M, Heissel J, Shen YC. Spatial analysis of access to psychiatrists for US military personnel and their families. *JAMA Netw Open.* 2023;6(1):e2249314. doi:10.1001/jamanetworkopen.2022.49314

eAppendix 1. Data and Methods to Define Relevant Geographic Access Area From Patient Perspective

eAppendix 2. Measurement of Gini Coefficient to Capture Income Inequality

eAppendix 3. Time Trends

eReferences

eTable 1. Logistic Regression Results Where Shortage Is Defined as <1 Psychiatrist per 30K Relevant Population

eTable 2. Logistic Regression Assuming Relevant Geographic Access Area Is Within 40-Min Drive

eTable 3. Logistic Regression Assuming Only 37% of Civilian Providers Accept TRICARE

This supplementary material has been provided by the authors to give readers additional information about their work.

© 2023 Bacolod M et al. *JAMA Network Open.*

eAppendix 1. Data and Methods to Define Relevant Geographic Access Area From Patient Perspective

We combine monthly data from January 2016 to September 2020 to form our analysis. First, we use the Defense Enrollment Eligibility Reporting System (DEERS) to capture beneficiary population size, demographic, and military branch of service characteristics of TRICARE beneficiaries for a given community. Second, we use the Medical Expense and Performance Reporting System (MEPRS) and the Defense Medical Human Resource System internet (DMHRSi) to capture MTF psychiatrist capacity; and the National Plan and Provider Enumeration System National Provider Identifier (NPI) data to identify civilian psychiatrists and their practice ZIP code. Third, we use the U.S. Census, American Community Survey (ACS), and the Social Determinants of Health Database to capture the community's overall population and socioeconomic characteristics.[17] Finally, we use a web-based query[18] to derive database of driving time between centers of each ZIP code community and (1) the MTF's and (2) ZIP code centers of civilian psychiatrists' practicing location.

We use ZIP codes to define community because it is the smallest geographic unit we can capture across data sources. However, the closest psychiatrist is not necessarily practicing in the same ZIP code as the patient, especially in cities, so we need to define a more robust geographic boundary to assess patients' access to psychiatrists. Rather than using the catchment area approach which defines relevant geographic boundaries from a facility's perspective, we define geographic coverage from the patient's perspective. For each community, we use travel time concept to define the relevant access area, rather than alternative definitions such as political boundaries (i.e., state or county lines) or fixed radius approach (such as the 20-mile radius catchment area used by the Defense Health Agency) for several reasons. First, using travel time does not impose arbitrary limits on actual access like political boundaries—an Idaho patient who

© 2023 Bacolod M et al. *JAMA Network Open.*

lives near the state line of Oregon would have easier access to a psychiatrist in a certain part of Oregon than in Idaho. Second, unlike a fixed-mile radius approach which cannot capture geographical barriers such as mountains and water bodies, the driving time approach captures geographic access more accurately and consistently. Third, travel time is positively associated with treatment attrition. For example, female veterans are more likely to attrite from Veteran's Heath Administration care the longer their drive time, particularly for new patients[1], and longer travel time is associated with fewer annual visits for depression treatment.[2]

Researchers have used 30 minutes of travel time as a benchmark for various medical treatments such as emergency caesarean delivery,[3] cardiac care,[4,5] and opioid treatment[6]; while others have used a linear measure of time and its relationship to care.[1,2,7–9] The U.S. Department of Health and Human Services uses 30 minutes travel time as the rational area of coverage for primary care and 40 minutes for dental and mental health care.[10] We chose 30 minutes car driving time (1-hour round trip) as a reasonable time threshold that TRICARE beneficiaries are willing to travel on a repeated basis since each mental health episode can require multiple follow-up visits. It should be noted that while driving time accounts for large percent of travel time, actual travel time would be longer than the 30-minute driving time due to various factors, such as wait time for the ride, navigating parking structure, and in the case of public transportation, frequent stops. In our sensitivity analysis, we use 40 minutes of driving time as alternative threshold.

Based on the above geographic access definition, we take the following steps to capture the collection of ZIP codes and MTFs with psychiatrists that are within a 30-minute driving time of a given community. First, for each ZIP code where we have a TRICARE beneficiary or civilian providers, we obtain longitude and latitude coordinates of the ZIP code interior center

© 2023 Bacolod M et al. *JAMA Network Open.*

based on the US Census. Second, for each MTF, we obtain longitude and latitude coordinates via automated web interface based on their physical address. Last, we derived a travel-time database using web-based queries that identify driving time under normal traffic conditions between each pair (ZIP code to ZIP code for care through a civilian provider and ZIP code to MTF for MTF-provided care). All psychiatrists practicing in ZIP codes and MTFs that can be reached within a 30-minute driving time from a given community would be considered geographically accessible to beneficiaries for that community.

Based on our geographic access definition, we derived a drive-time database using web-based queries that identify driving time under normal traffic conditions between each pair based on their longitude and latitude coordinates (ZIP code to ZIP code for care through a civilian provider and ZIP code to MTF for MTF-provided care).[18] All psychiatrists practicing in ZIP codes and MTFs that can be reached within a 30-minute driving time from a given community would be considered geographically accessible to beneficiaries for that community.

© 2023 Bacolod M et al. *JAMA Network Open.*

eAppendix 2. Measurement of Gini Coefficient to Capture Income Inequality

The Gini coefficient we employ measures the extent of income inequality in a ZIP code community. More generally the Gini coefficient is a measure of statistical dispersion or variation in the distribution of income within some population, a nation, or in our case, a community. Rather than metrics such as standard deviation which may be difficult to interpret, the Gini coefficient is expressed as an index ranging from 0 (perfect equality) to 1 (complete inequality). Actual values of 0 or 1 are very rare. The calculation involves measuring how far a population's actual distribution of income is from a hypothetical distribution where all individuals in the population earned equal income. Summing up (or integrating) these distances and scaling to be in the [0,1] range yields the Gini coefficient. For instance, consider two communities. The first community is comprised of middle-class households with roughly similar incomes. Suppose the second community's income distribution is bimodal, where significant proportions of households have either particularly high or particularly low incomes. The distance of this second community's actual income distribution from a hypothetical equal-income distribution (Gini coefficient) will be higher than the first community's.

© 2023 Bacolod M et al. *JAMA Network Open.*

eAppendix 3. Time Trends

Figure below shows the temporal trend in TRICARE populations who reside in communities that are psychiatrist shortage areas in both MTF and civilian sectors. The active-duty population typically resides near MTFs, and 11-17% of active duty populations are in communities with a shortage of both MTF and civilian psychiatrists. Non-active duty populations are spread across a more diverse set of communities, and almost 40% of this subpopulation does not have adequate access to either type of psychiatrist. The fraction of TRICARE beneficiaries residing in shortage areas remain similar over time. The active duty population has a small upward trend from 11% in January 2016 to 17% in September 2020.

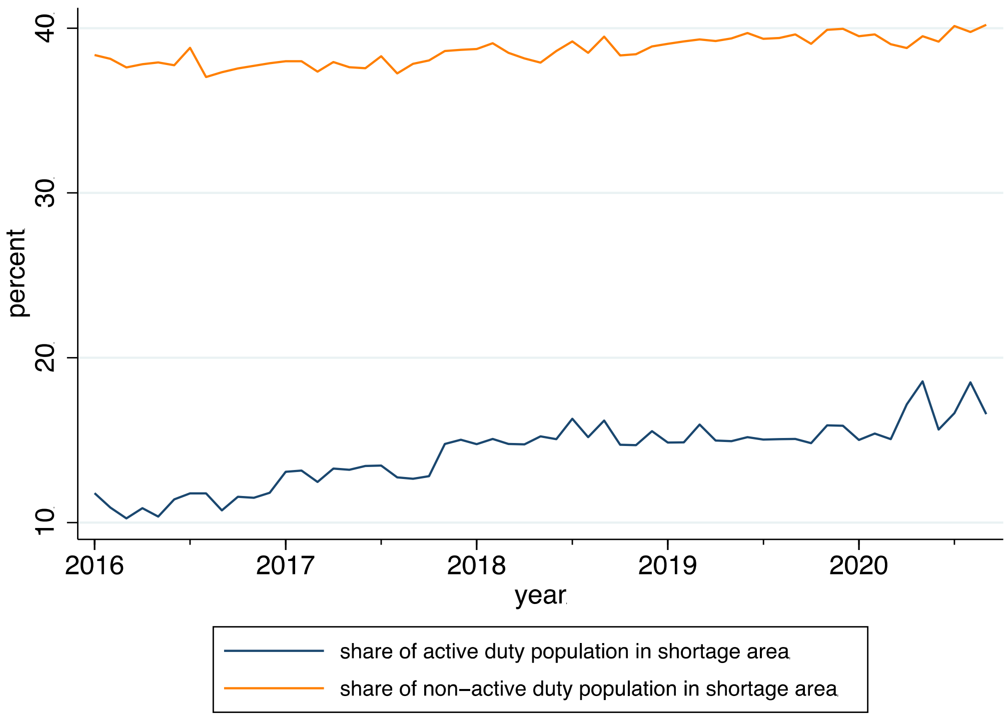

© 2023 Bacolod M et al. *JAMA Network Open.*

eReferences

1. Friedman SA, Frayne SM, Berg E, et al. Travel time and attrition from VHA care among women Veterans: How far is too far? *Med Care*. 2015;53(4 0 1):S15-S22. doi:10.1097/MLR.0000000000000296

2. Fortney J, Rost K, Zhang M, Warren J. The Impact of Geographic Accessibility on the Intensity and Quality of Depression Treatment. *Medical Care*. 1999;37(9):884-893.

3. Uribe-Leitz T, Matsas B, Dalton MK, et al. Geospatial Analysis of Access to Emergency Cesarean Delivery for Military and Civilian Populations in the US. *JAMA Network Open*. 2022;5(1):e2142835. doi:10.1001/jamanetworkopen.2021.42835

4. Sommerhalter KM, Insaf TZ, Akkaya-Hocagil T, et al. Proximity to Pediatric Cardiac Surgical Care among Adolescents with Congenital Heart Defects in 11 New York Counties. *Birth Defects Research*. 2017;109(18):1494-1503. doi:10.1002/bdr2.1129

5. Graves BA. Geographic Analysis of Cardiac Interventional Services in Alabama. *Journal of Cardiovascular Nursing*. 2011;26(4):E1. doi:10.1097/JCN.0b013e3181ecaacb

6. Hyder A, Lee J, Dundon A, et al. Opioid Treatment Deserts: Concept development and application in a US Midwestern urban county. *PLOS ONE*. 2021;16(5):e0250324. doi:10.1371/journal.pone.0250324

7. Chan L, Hart LG, Goodman DC. Geographic Access to Health Care for Rural Medicare Beneficiaries. *The Journal of Rural Health*. 2006;22(2):140-146. doi:10.1111/j.1748-0361.2006.00022.x

8. Lee CS, Morris A, Van Gelder RN, Lee AY. Evaluating Access to Eye Care in the Contiguous United States by Calculated Driving Time in the United States Medicare Population. *Ophthalmology*. 2016;123(12):2456-2461. doi:10.1016/j.ophtha.2016.08.015

9. Kleinman RA. Comparison of Driving Times to Opioid Treatment Programs and Pharmacies in the US. *JAMA Psychiatry*. 2020;77(11):1163-1171. doi:10.1001/jamapsychiatry.2020.1624

10. *42 CFR Part 5 -- Designation of Health Professional(s) Shortage Areas*. Vol 45 FR 76000.; 1980. Accessed April 28, 2022. https://www.ecfr.gov/current/title-42/chapter-I/subchapter-A/part-5

© 2023 Bacolod M et al. *JAMA Network Open.*

eTable 1. Logistic Regression Results Where Shortage Is Defined as <1 Psychiatrist per 30K Relevant Population

	Shortage area for both MTF and civilian psychiatrists	
	Odds ratio from single dimension models [95% CI]	**Model 6**: Fully regression- adjusted odds ratio
**Model 1: Socioeconomic conditions**		
mean income without high income inequality (ref)	1.00	1.00
low income with high inequality	2.55**	1.79**
	[2.00,3.26]	[1.39,2.31]
low income without high inequality	1.08	1.44*
	[0.81,1.45]	[1.08,1.90]
mean income with high inequality	1.19	0.93
	[0.94,1.52]	[0.72,1.20]
high income without high inequality	0.49**	0.45**
	[0.36,0.67]	[0.33,0.62]
high income with high inequality	0.32**	0.30**
	[0.24,0.44]	[0.22,0.41]
**Model 2: Regions and rurality**		
Urban community (ref)	1.00	1.00
Rural community	5.32**	5.59**
	[4.11,6.89]	[4.34,7.21]
South (ref)	1.00	1.00
Alaska	0.77	1.02
	[0.39,1.49]	[0.47,2.21]
Hawaii	0.36**	0.30**
	[0.30,0.44]	[0.19,0.46]
Midwest	0.61**	0.49**
	[0.45,0.84]	[0.35,0.69]
Northeast	0.37**	0.35**
	[0.26,0.52]	[0.25,0.49]
West	0.73+	0.65*
	[0.51,1.05]	[0.43,0.97]
**Model 3: Race and ethnicity distribution**		
Share of population that are Black (low=ref)	1.00	1.00
Medium share	0.75**	0.78*
	[0.62,0.91]	[0.63,0.98]
High share	0.68**	0.49**
	[0.52,0.89]	[0.36,0.68]
Share of population that are Hispanic (low=ref)	1.00	1.00
Medium share	0.65**	0.85
	[0.54,0.79]	[0.69,1.05]
High share	0.64**	0.69+
	[0.47,0.86]	[0.47,1.03]

© 2023 Bacolod M et al. *JAMA Network Open.*

**Model 4: Primary service branch of beneficiaries**		
Mixed service community (ref)	1.00	1.00
Army	1.22	1.08
	[0.95,1.56]	[0.87,1.33]
Navy	0.50*	0.65
	[0.27,0.94]	[0.34,1.26]
USMC	0.51**	0.49*
	[0.34,0.78]	[0.27,0.89]
Air Force	0.68*	0.79
	[0.50,0.93]	[0.54,1.18]
**Model 5: Presence and type of beneficiaries**		
Share of population that are TRICARE (low=ref)	1.00	1.00
Medium share	0.98	1.24+
	[0.78,1.22]	[0.98,1.57]
High share	0.52**	0.73
	[0.35,0.78]	[0.45,1.16]
Share of TRICARE that are dependent children (low=ref)	1.00	1.00
Medium share	1.10	1.08
	[0.85,1.42]	[0.84,1.41]
High share	1.44*	1.32
	[1.07,1.92]	[0.93,1.86]
Share of TRICARE that are retirees (low=ref)	1.00	1.00
Medium share	1.12	1.20
	[0.77,1.64]	[0.78,1.84]
High share	1.73**	1.63*
	[1.19,2.52]	[1.04,2.54]
Share of TRICARE that are other non-active duty (low=ref)	1.00	1.00
Medium share	1.05	0.92
	[0.76,1.43]	[0.68,1.25]
High share	0.88	0.68*
	[0.66,1.17]	[0.51,0.91]
N (community-years)	2,098,124	

Note: * p< 0.05, **p<0.01. Additional variables common in all models include year dummies, community population (log transformed). Robust standard errors clustered a ZIP code community and county levels.

© 2023 Bacolod M et al. *JAMA Network Open.*

eTable 2. Logistic Regression Assuming Relevant Geographic Access Area Is Within 40-Min Drive

	**Shortage area for both MTF and civilian psychiatrists**		**No MTF nor civilian psychiatrists within 40-min**	
	Odds ratio from single dimension models [95% CI]	**Model 6**: Fully regression-adjusted odds ratio	Odds ratio from single dimension models	**Model 6**: Fully regression-adjusted odds ratio
**Model 1: Socioeconomic conditions**				
mean income without high income inequality (ref)	1.00	1.00	1.00	1.00
low income with high inequality	2.73** [2.16,3.46]	2.21** [1.70,2.88]	3.08** [2.31,4.12]	2.36** [1.31,4.24]
low income without high inequality	0.97 [0.70,1.34]	1.36+ [0.99,1.85]	1.49+ [0.93,2.38]	1.58 [0.74,3.37]
mean income with high inequality	2.23** [1.74,2.86]	1.97** [1.51,2.57]	1.54** [1.14,2.08]	1.40 [0.81,2.42]
high income without high inequality	0.64** [0.47,0.88]	0.62** [0.46,0.84]	0.42** [0.28,0.64]	0.75 [0.46,1.21]
high income with high inequality	0.58** [0.42,0.80]	0.63** [0.46,0.86]	0.53** [0.35,0.80]	0.74 [0.42,1.32]
**Model 2: Regions and rurality**				
Urban community (ref)	1.00	1.00	1.00	1.00
Rural community	4.13** [3.26,5.24]	9.01** [6.41,12.68]	24.19** [13.01,44.98]	10.53** [6.63,16.71]
South (ref)	1.00	1.00	1.00	1.00
Alaska	0.35 [0.09,1.38]	0.48 [0.13,1.83]	3.21* [1.22,8.47]	7.53** [2.72,20.85]
Hawaii	0.33** [0.26,0.42]	0.29** [0.19,0.44]	9.39** [7.20,12.25]	20.71** [8.48,50.55]
Midwest	0.75+ [0.55,1.02]	0.86 [0.58,1.27]	0.92 [0.63,1.34]	0.73+ [0.50,1.06]
Northeast	0.38** [0.27,0.55]	0.54** [0.35,0.83]	0.50** [0.30,0.83]	0.32** [0.18,0.54]
West	0.72 [0.48,1.07]	0.66+ [0.40,1.08]	3.23** [2.18,4.78]	4.19** [2.83,6.21]
**Model 3: Race and ethnicity distribution**				
Share of population that are Black (low=ref)	1.00	1.00	1.00	1.00
Medium share	0.82* [0.67,0.99]	0.85 [0.69,1.04]	0.40** [0.23,0.71]	1.03 [0.59,1.78]
High share	0.87 [0.66,1.14]	0.67* [0.48,0.94]	0.15** [0.11,0.22]	0.51** [0.34,0.76]
Share of population that are Hispanic (low=ref)	1.00	1.00	1.00	1.00
Medium share	0.86 [0.69,1.06]	1.21+ [0.97,1.51]	0.96 [0.71,1.29]	0.92 [0.72,1.19]
High share	0.58** [0.41,0.82]	0.77 [0.51,1.17]	1.37 [0.81,2.31]	1.30 [0.72,2.35]

© 2023 Bacolod M et al. *JAMA Network Open.*

**Model 4: Primary service branch of beneficiaries**				
Mixed service community (ref)	1.00	1.00	1.00	1.00
Army	1.20	1.06	1.05	1.10
	[0.91,1.57]	[0.82,1.38]	[0.70,1.57]	[0.83,1.48]
Navy	0.66	0.72	0.36*	0.67
	[0.34,1.28]	[0.36,1.44]	[0.16,0.82]	[0.29,1.55]
USMC	0.90	0.97	0.36	0.30
	[0.55,1.48]	[0.45,2.09]	[0.07,1.86]	[0.03,3.27]
Air Force	0.88	1.15	0.46**	0.52*
	[0.63,1.23]	[0.75,1.75]	[0.26,0.81]	[0.28,0.94]
**Model 5: Presence and type of beneficiaries**				
Share of population that are TRICARE (low=ref)	1.00	1.00	1.00	1.00
Medium share	0.89	1.11	0.78+	0.97
	[0.70,1.13]	[0.85,1.44]	[0.61,1.00]	[0.78,1.22]
High share	0.86	1.26	0.47+	0.35
	[0.57,1.28]	[0.81,1.97]	[0.21,1.02]	[0.09,1.39]
Share of TRICARE that are dependent children (low=ref)	1.00	1.00	1.00	1.00
Medium share	1.17	0.97	0.45**	0.99
	[0.96,1.43]	[0.80,1.17]	[0.35,0.60]	[0.73,1.36]
High share	1.49**	1.04	0.77	2.08
	[1.18,1.88]	[0.80,1.35]	[0.34,1.77]	[0.70,6.24]
Share of TRICARE that are retirees (low=ref)	1.00	1.00	1.00	1.00
Medium share	1.31	1.46+	1.54	1.89
	[0.86,2.01]	[0.95,2.22]	[0.66,3.62]	[0.86,4.18]
High share	1.37	1.43+	4.02**	2.90+
	[0.92,2.05]	[0.94,2.19]	[2.46,6.58]	[0.98,8.56]
Share of TRICARE that are other non-active duty (low=ref)	1.00	1.00	1.00	1.00
Medium share	2.35**	1.76**	0.55**	1.10
	[1.73,3.20]	[1.34,2.33]	[0.40,0.74]	[0.71,1.69]
High share	2.13**	1.33+	0.49**	1.12
	[1.54,2.95]	[0.99,1.78]	[0.36,0.65]	[0.81,1.55]
N (community-years)	2,098,124			

Note: * p< 0.05, **p<0.01. Additional variables common in all models include year dummies, community population (log transformed). Robust standard errors clustered a ZIP code community and county levels.

© 2023 Bacolod M et al. *JAMA Network Open.*

eTable 3. Logistic Regression Assuming Only 37% of Civilian Providers Accept TRICARE

	**Shortage area for both MTF and civilian psychiatrists**		**No MTF nor civilian psychiatrists within 30-min**	
	Odds ratio from single dimension models [95% CI]	**Model 6**: Fully regression-adjusted odds ratio	Odds ratio from single dimension models	**Model 6**: Fully regression-adjusted odds ratio
**Model 1: Socioeconomic conditions**				
mean income without high income inequality (ref)	1.00	1.00	1.00	1.00
low income with high inequality	1.95** [1.40,2.72]	1.19 [0.87,1.62]	3.18** [2.52,4.01]	2.09** [1.49,2.94]
low income without high inequality	0.75+ [0.54,1.06]	1.26 [0.93,1.71]	1.36* [1.03,1.80]	1.60* [1.06,2.43]
mean income with high inequality	1.57** [1.17,2.10]	0.99 [0.74,1.33]	1.25+ [0.98,1.58]	0.95 [0.70,1.29]
high income without high inequality	0.88 [0.65,1.19]	0.63** [0.49,0.82]	0.35** [0.26,0.46]	0.44** [0.31,0.61]
high income with high inequality	0.64** [0.46,0.88]	0.43** [0.32,0.58]	0.44** [0.32,0.61]	0.45** [0.31,0.67]
**Model 2: Regions and rurality**				
Urban community (ref)	1.00	1.00	1.00	1.00
Rural community	2.39** [1.75,3.26]	5.02** [3.55,7.12]	18.65** [13.50,25.77]	8.69** [6.54,11.55]
South (ref)	1.00	1.00	1.00	1.00
Alaska	0.45+ [0.19,1.05]	0.73 [0.22,2.44]	3.95** [1.82,8.56]	7.77** [2.44,24.73]
Hawaii	0.09** [0.06,0.14]	0.05** [0.03,0.09]	1.55** [1.26,1.92]	1.75+ [0.92,3.31]
Midwest	0.99 [0.71,1.39]	0.71 [0.46,1.08]	0.69+ [0.47,1.03]	0.45** [0.33,0.62]
Northeast	0.55** [0.40,0.77]	0.45** [0.31,0.67]	0.50** [0.33,0.76]	0.27** [0.18,0.40]
West	0.88 [0.52,1.47]	0.80 [0.39,1.64]	1.40 [0.92,2.13]	1.61* [1.08,2.41]
**Model 3: Race and ethnicity distribution**				
Share of population that are Black (low=ref)	1.00	1.00	1.00	1.00
Medium share	0.74* [0.55,0.98]	0.82 [0.63,1.06]	0.52** [0.40,0.69]	0.75 [0.53,1.07]
High share	0.64* [0.45,0.93]	0.58* [0.35,0.95]	0.33** [0.25,0.43]	0.43** [0.32,0.59]
Share of population that are Hispanic (low=ref	1.00	1.00	1.00	1.00
Medium share	0.58** [0.45,0.76]	0.83 [0.66,1.04]	0.64** [0.52,0.79]	0.89 [0.69,1.15]
High share	0.47** [0.28,0.78]	0.50* [0.27,0.93]	0.54** [0.37,0.80]	0.66+ [0.43,1.04]

© 2023 Bacolod M et al. *JAMA Network Open.*

**Model 4: Primary service branch of beneficiaries**				
Mixed service community (ref)	1.00	1.00	1.00	1.00
Army	0.78	1.02	1.19	1.12
	[0.58,1.05]	[0.78,1.33]	[0.92,1.54]	[0.93,1.35]
Navy	0.32**	0.45+	0.43*	0.79
	[0.16,0.66]	[0.20,1.02]	[0.22,0.83]	[0.40,1.56]
USMC	0.39**	0.60	0.49	0.53
	[0.20,0.77]	[0.21,1.71]	[0.21,1.17]	[0.10,2.79]
Air Force	0.55**	0.92	0.56**	0.62+
	[0.40,0.75]	[0.58,1.48]	[0.39,0.82]	[0.36,1.07]
**Model 5: Presence and type of beneficiaries**				
Share of population that are TRICARE (low=ref)	1.00	1.00	1.00	1.00
Medium share	0.75+	0.88	0.84	1.14
	[0.56,1.02]	[0.66,1.18]	[0.69,1.04]	[0.93,1.41]
High share	0.38**	0.59*	0.47**	0.48*
	[0.27,0.52]	[0.38,0.93]	[0.30,0.75]	[0.24,0.95]
Share of TRICARE that are dependent children (low=ref)	1.00	1.00	1.00	1.00
Medium share	1.53**	1.03	0.55**	0.98
	[1.16,2.01]	[0.80,1.33]	[0.46,0.65]	[0.75,1.27]
High share	1.59**	0.82	0.77	1.77+
	[1.16,2.20]	[0.58,1.16]	[0.52,1.16]	[0.95,3.28]
Share of TRICARE that are retirees (low=ref)	1.00	1.00	1.00	1.00
Medium share	1.67**	1.60*	1.68*	2.17*
	[1.18,2.36]	[1.09,2.34]	[1.08,2.63]	[1.20,3.92]
High share	2.07**	1.95**	3.74**	3.41**
	[1.38,3.08]	[1.22,3.14]	[2.54,5.51]	[1.91,6.07]
Share of TRICARE that are other non-active duty (low=ref)	1.00	1.00	1.00	1.00
Medium share	1.25	0.82	0.58**	0.89
	[0.96,1.62]	[0.61,1.10]	[0.47,0.72]	[0.65,1.22]
High share	0.96	0.57**	0.53**	0.83
	[0.66,1.39]	[0.40,0.81]	[0.43,0.67]	[0.61,1.14]
N (community-years)	2,098,124			

Note: * p< 0.05, **p<0.01. Additional variables common in all models include year dummies, community population (log transformed). Robust standard errors clustered a ZIP code community and county levels.

© 2023 Bacolod M et al. *JAMA Network Open.*
