# Supplementary material for: Spatial Analysis of Access to Psychiatrists for US Military Personnel and Their Families
Source: JAMA Netw Open. 2023 Jan 3;6(1):e2249314. doi: 10.1001/jamanetworkopen.2022.49314 (PMC9856908; doi:10.1001/jamanetworkopen.2022.49314)
Supplement: Supplement 2. — Data Sharing Statement [file jamanetwopen-e2249314-s002.pdf]

Data Sharing Statement

Bacolod. Spatial Analysis of Access to Psychiatrists for US Military Personnel and Their Families. *JAMA Netw Open*. Published January 03, 2023.
doi:10.1001/jamanetworkopen.2022.49314

Data
Data available: No

Additional Information
Explanation for why data not available: Data are owned by the Defense Health Agency. Researchers who wish to use the data would need to obtain data sharing agreement with data owner.
